# Mechanisms underpinning interventions to reduce sexual violence in armed conflict: A realist-informed systematic review

**DOI:** 10.1186/s13031-015-0047-4

**Published:** 2015-07-13

**Authors:** Jo Spangaro, Chinelo Adogu, Anthony B. Zwi, Geetha Ranmuthugala, Gawaine Powell Davies

**Affiliations:** School of Social Sciences, University of New South Wales, Ground Floor Morven Brown Building, Sydney, 2052 NSW Australia; School of Rural Medicine, The University of New England, Armidale, Australia; Centre for Primary Health Care and Equity, University of New South Wales, Sydney, Australia

**Keywords:** Sexual violence, Conflict and crisis-related sexual violence, Armed conflict, Humanitarian crisis, Interventions, Realist approach, Systematic review

## Abstract

Sexual violence is recognised as a widespread consequence of armed conflict and other humanitarian crises. The limited evidence in literature on interventions in this field suggests a need for alternatives to traditional review methods, particularly given the challenges of undertaking research in conflict and crisis settings. This study employed a realist review of the literature on interventions with the aim of identifying the mechanisms at work across the range of types of intervention. The realist approach is an exploratory and theory-driven review method. It is well suited to complex interventions as it takes into account contextual factors to identify mechanisms that contribute to outcomes. The limited data available indicate that there are few deterrents to sexual violence in crises. Four main mechanisms appear to contribute to effective interventions: increasing the risk to offenders of being detected; building community engagement; ensuring community members are aware of available help for and responses to sexual violence; and safe and anonymous systems for reporting and seeking help. These mechanisms appeared to contribute to outcomes in multiple-component interventions, as well as those relating to gathering firewood, codes of conduct for personnel and legal interventions. Drawing on pre-existing capacity or culture in communities is an additional mechanism which should be explored. Though increasing the risk to offenders of being detected was assumed to be a central mechanism in deterring sexual violence, the evidence suggests that this mechanism operated only in interventions focused on gathering firewood and providing alternative fuels. The other three mechanisms appeared important to the likelihood of an intervention being successful, particularly when operating simultaneously. In a field where robust outcome research remains likely to be limited, realist methods provide opportunities to understand existing evidence. Our analysis identifies the important potential of building in mechanisms involving community engagement, awareness of responses and safe reporting provisions into the range of types of intervention for sexual violence in crises.

## Background

Sexual violence is now well recognised to be a consequence of armed conflict [1–9], with a recent global summit convened by the British Foreign Secretary attended by delegates from 123 countries. Twenty one per cent of female refugees and internally displaced people in complex humanitarian emergencies have experienced sexual violence according to a recent review across 14 countries [10]. We define sexual violence as “sexual acts committed against a person, or in which a person is caused to engage by force, threat of force or coercion such as that caused by fear of violence, duress, detention, psychological oppression or abuse of power, or by taking advantage of a coercive environment or a person’s incapacity to give genuine consent” [11]. ‘Conflict’ here refers to armed conflict between state or other militias, excluding civil disorder and terrorism. Other humanitarian crises, particularly natural disasters, involve many of the same risk factors for sexual violence as armed conflict, particularly displacement, breakdown of social and legal systems, and dependence on humanitarian workers or security forces [12, 13], and warrant consideration along with conflict settings in terms of this issue. Sexual violence during armed conflict is a public health as well as a security and justice issue [14] with significant leadership being demonstrated by the World Health Organization [14, 15]. Its intervention recognises the significant short and long-term health effects, which include injury, HIV, sexually transmitted infections, unwanted pregnancies, traumatic fistulae, depression, post-traumatic stress disorder, anxiety, stigma and social rejection.

Recent years have seen rapid expansion in programs across different sectors to address conflict and crisis-related sexual violence [16, 17]. We identify seven types of intervention: i) provision of care for survivors of sexual violence; ii) initiatives to enhance livelihoods of women and so provide protection from sexual exploitation and abuse; iii) community mobilisation strategies to increase awareness and mutual protection; iv) initiatives directed at personnel providing services or protection (such as gender-specific recruitment of security staff and codes of conduct for military, security or aid workers prohibiting sexual contact with community members); v) systems and security responses (such as firewood patrols or segregated washing facilities in refugee camps); vi) legal interventions; and vii) multiple-component interventions which integrate two or more of these strategies. There is an urgent need to gauge the impact of these interventions, while the interactions between these types of intervention, for example the need for medical evidence to be gathered in order for prosecution to occur, point to the value of a broad view.

Previously we reported on a narrative systematic review of the evidence for reduced risk or incidence of sexual violence from the range of intervention types [18]. The most positive outcomes were linked to interventions with multiple components and where community engagement had occurred. However, few details of interventions were provided and outcome studies were generally of limited quality. A separate systematic review on health interventions in humanitarian crises or conflict concurred that evidence in respect of gender-based violence (GBV) is limited, calling for more information on the context of GBV and new methodological approaches to understanding this problem [19]. A third review focusing on mental health and psychosocial support interventions after sexual and other forms of GBV in armed conflicts tentatively concluded that benefits occurred, though noted that robust conclusions on the effectiveness of particular approaches were not possible [20].

The paucity of evidence in this area does not, we believe, reflect indifference. The challenges of undertaking research in crisis settings, particularly on a topic as sensitive as sexual violence, are considerable [21] and it remains likely that the canon of evidence on interventions will be slow to build. There is merit in an alternative approach which involves analysis of existing studies to identify both mechanisms which underpin successful interventions as well as the contextual factors which are required for these mechanisms to operate. Realist methods offer such an opportunity, providing a path to making use of the limited data on interventions in the field.

The realist approach to research recognises that social problems and therefore interventions to address them are complex [22]. It is increasingly used to study complex interventions [23] where traditional approaches to evaluation or review, which determine solely whether or not an intervention works, have been less productive [22, 24]. Traditional systematic reviews such as those undertaken with the Cochrane Collaboration, generally privilege evidence gathered by means of randomised control trials. However, there is now greater recognition that a wide variety of study designs are required to evaluate public health interventions, with no single method able to answer all relevant questions about effectiveness [25]. Realist review methodology has been applied to primary research as well as systematic reviews on the evidence for interventions as diverse as: retention of health workers in remote areas [26] and implementation of routine screening for intimate partner violence [27]. The approach is applicable here as sexual violence in conflict and crisis is a complex social and legal problem.

A realist approach highlights the importance of the context in which interventions are implemented and the different levels at which they operate. The impact of interventions depends on the social and cultural context within which they are introduced, with the result that the same interventions can have different outcomes in different contexts. The approach also aims to understand an intervention’s underlying theory of change by postulating mechanisms [28] which are triggered by the interaction of the context and the intervention. These theories are then tested and refined in the synthesis process [28], illuminating the possibilities for applying the same mechanism to other interventions and to different contexts.

We build on our earlier narrative analysis of identified studies [18] and report here on the realist analysis where we aimed to answer the question: what are the underlying mechanisms by which these interventions appeared to bring about reduced risk or incidence of sexual violence? Recognising the effectiveness of multiple-component interventions, we focus first on the mechanisms that seem to underpin these, and then those evident in stand-alone interventions, where sufficient data exists.

## Methods

Realist reviews take an exploratory, interpretive approach to examine the links between the contextual factors and mechanisms that contribute to outcomes [23], and as such are different from many other reviews [29]. Mechanisms are “underlying entities, processes or structures which operate in particular contexts to generate outcomes of interest” [24] (p 2). The realist approach identifies the mechanisms involved in an intervention to understand how it is intended to work [28] then tests these mechanisms against the empirical evidence of outcomes [22, 24, 29].

Similar to the PRISMA guidelines for reporting traditional systematic reviews, the RAMESES standards guide reporting of realist systematic reviews [24]. The methods used are reported in line with the four stages of the review as outlined in these guidelines, that is: i) exploratory scoping; ii) proposal of theory; iii) data search and extraction; and iv) data synthesis.

### Exploratory scoping

A nine member advisory group met monthly to provide oversight through the life of the project. Representation included senior policy makers with responsibility for this policy area from the World Health Organization and the International Planned Parenthood Foundation, former and current practitioners involved in oversight of sexual violence responses in conflict and crisis settings in the African and Asian sub-continents and Pacific region, as well as two academics involved in research on legal responses to conflict related sexual violence. This group helped refine the questions and scope of the study, and advised on areas of focus of most utility to the field.

### Proposal of theory and definitions

Through a series of workshops, hypothetical ‘context-mechanism-outcome’ configurations were developed. These configurations set out the potential path of how outcomes are brought about, from the perspective of those involved. They identify the mechanism in question as well as contextual factors thought to be required to trigger the mechanism, and outcomes resulting from this activation. Possible configurations comprising potential contextual factors, mechanisms and likely outcomes were hypothesised for each of the seven identified types of intervention. The proposed mechanisms are listed in Table 1 along with intervention types in which each mechanism is anticipated to operate. This theorising was undertaken by the team and two members of the advisory group (CR, SRH), informed by both our practice experience and the literature. The six key mechanisms we proposed are reported in the first section of the results. Specific contextual factors were hypothesised as relevant for each intervention type. As an example, those for systems and security interventions included whether: priority was given to allocation of resources; risk assessment of possible perpetrator opportunities was undertaken; and agencies monitored implementation of the intervention (The full configurations are available from the authors).

**Table 1:** Types of intervention for sexual violence and proposed underpinning mechanisms and outcomes

	Type of intervention	Definition	Proposed mechanisms	Proposed outcomes
Individual	Survivor care responses	Medical, psycho-social care & advocacy for survivors	1	Prosecution enabled through survivor reports, deterring sexual violence
			3	Harm from sexual violence is reduced
			4	Women seek help
	Livelihood strategies	Micro-finance and/or training to increase women’s independence pre/post sexual violence	5	Sexual exploitation and abuse is reduced
				Harm from sexual violence is reduced
Community	Community mobilisation	Education of rights in regard to sexual coercion; increased opportunities for women to participate in political, economic and social activities	1, 2, 3, 4, 5	Sexual violence is reduced
			4, 5	Survivors get help and report
			6	Community protects women & sanctions sexual violence
Societal	Personnel interventions	Protocols with military/peacekeepers/aid workers to reduce sexual exploitation and abuse; recruitment of female officers	1	Sexual violence reduced
			3, 5	Survivors feel safe to report incidents
	Systems and security interventions	Patrols or firewood/fuel distribution to reduce vulnerability to sexual violence	1	Sexual violence is reduced
	Legal strategies	Specialist prosecution units/tribunals; customary justice systems; International Criminal Court indictments	1	Sexual violence is reduced as a result of deterrence through arrest/action/conviction
			4, 5	Survivors feel safe to report incidents
	Multiple component interventions	Integration of any two or more of the above strategies	As per individual strategies employed	As per individual strategies employed

1. Rape is risky; 2. Rape is unacceptable; 3. There is help for this problem; 4. It’s safe to tell; 5. We have rights; 6. We can work together to address this problem

Following the initial theorisation, the team identified a middle range or ‘candidate’ theory that underpinned these hypothesised mechanisms, which could be tested by examining existing evidence [22]. We drew on theory of deterrence from the field of criminology, which holds that the likelihood of committing a crime depends on the offender’s perceptions of the chance of detection [30]. Crimes are much more likely to be committed if the offender believes there is a low chance of being detected or held to account. Contrary to popular belief, this has more influence than the severity of punishment [30]. Accordingly we hypothesised that sexual violence is reduced when perpetrators perceive there is a high likelihood of detection or being held to account, which may include prosecution. We also adopted an ecological framework which identifies factors contributing to GBV at the individual, familial and societal levels [31]. We identified that interventions are applied at each of these four levels and grouped them in this way in the analysis (see Table 1). Our assumption was that at the societal level unequal gender relations would be a key factor contributing to sexual violence [7, 32, 33]. During the analysis we remained alert for unanticipated mechanisms and additional contextual factors, such as country or type of conflict, which appeared to influence outcomes.

### Data search, extraction and appraisal

As with any form of systematic review, rigorous documented searching was undertaken according to defined terms and specified inclusion criteria. We conducted an extensive literature search using 23 bibliographic databases^1^ using the terms ‘sexual violence’ and ‘armed conflict’ or ‘humanitarian crisis’ and their synonyms, dependent on database terms.^2^ We also searched 26 websites and manually searched three journals: *Violence Against Women; Medicine, Conflict and Survival*; and *Disasters*. Inclusion criteria were primary empirical data describing the impact of interventions aimed at reducing risk or incidence, or addressing harm from sexual violence occurring in conflict, post-conflict or other humanitarian crisis settings in low or middle-income countries, published between 1 January 1990 and 1 September 2011.

Data extracted from the studies included the country of intervention, nature of crisis, type of sexual violence (i.e. militarised, opportunistic, sexual exploitation and abuse, or exacerbated community violence), intervention type, (intervention) activities, target population (including age and sex of survivors of sexual violence), study methods, study group, outcome measures, results, and evidence of reduced incidence or risk. Indicators for reduced risk were developed as part of a conceptual framework for the study and based on recommendations for evaluating gender-based violence initiatives [34]. These indicators included: evidence of increased sense of safety in the community; improved wellbeing as a result of survivor programs; and awareness of rights by community members. Data extraction was assisted by EPPI-Centre software, EPPI Reviewer. Finally, study quality was appraised in relation to soundness of method, appropriateness of study type and relevance to review question, giving rise to an overall weight of evidence rating for each study [35, 36] (reported in Table 2).

**Table 2:** Overview of 20 studies reporting outcomes used in the analysis

Study	Intervention type	Country	Intervention components	Study design	Weight of evidence
Gruber (2005) [54]	Survivor care	Eritrea	Medical and counselling assistance to SV survivors	Qualitative interviews	Medium-Low
Hustache et al. (2009) [39]	Survivor care	Congo	Post-rape psychological support (median 2 sessions) following medical assessment/treatment	Baseline, follow-up survey	Medium
Manneschmidt & Griese (2009) [64]	Survivor care	Afghanistan	Psycho-social group counselling for Afghan women affected by war and domestic violence	Qualitative focus group	Low
Zraly & Nyirazinyoye (2010) [66]	Survivor care	Rwanda	Mutual support through advocacy/self-help groups	Qualitative interviews	Medium-Low
Denov (2006) [61]^a^	Livelihood	Sierra Leone	Disarmament, demobilisation and reintegration (DDR)	Field visits	Medium-Low
Jennings (2008) [58]	Personnel	Haiti &Liberia	Zero-tolerance policy for sexual eploitation and abuse; Code of Conduct (CoC) training; curfews; staff in uniforms at all times; fraternising discouraged; reporting hotline	Field visits	Medium-Low
Lattu (2008) [59]	Personnel	Kenya, Namibia & Thailand	Zero-tolerance policy for sexual exploitation and abuse; training on CoC; complaint boxes; community education;community participation in producing prevention film (Kenya only)	Qualitative interviews	Medium-High
CASA Consulting (2001) [55]	Systems & security	Kenya	Distribution of firewood in Dadaab refugee camp	Cross-sectional survey & field visits	Medium-High
Women’s Commission for Refugee Women and Children (2006) [57]	Systems & security	Sudan	Provision of fuel efficient stoves, alternative fuels and firewood patrols	Qualitative interviews & focus group	Medium-Low
Bizarri (2010) [56]	Systems & security	Kenya	Provision of firewood & fuel-efficient stoves; establishment of reporting mechanism	Multiple data case studies	Medium
Blogg et al. (2004) [38]	Multiple component interventions (MCIs)	Uganda & Congo	Survivor care: Medical/counselling support/legal information for survivors	Field visits	Medium-High
			Community mobilization: Engagement of community leaders; community alcohol ban, curfew & night patrols		
Schei & Dahl (1999) [53]	MCIs	Bosnia & Herzegovina	Survivor care: Recreational/craft group compared to weekly psychotherapy group (3–4 months)	Comparison two models + baseline, follow up survey	Low
			Personnel: Counsellor training		
Women’s Commission Refugee for Women and Children (2009) [52]	MCIs	Ethiopia	Livelihood strategies for refugee women	Qualitative interviews	Low
			Community mobilisation: GBV community discussions		
			Systems & security: Provision of ethanol stoves		
UNHCR (1997) [50]	MCIs	Tanzania	Survivor care: First response to survivors provided by trained volunteers	Field visits and interviews	Low
			Community mobilization: Consultation/awareness raising		
			Systems & security: Firewood patrols & distribution		
UNHCR (1998) [51]	MCIs	Tanzania	Survivor care: Medical treatment, advice and support	Implementation/description data	Low
			Community mobilization: Comm. awareness raising and problem solving		
			Systems & security: Increased police presence and communication Personnel: Community and health worker training		
Brouneus (2008) [60]	Legal	Rwanda	Rwanda Gacaca Courts (local village tribunals adapted to address war crimes)	Qualitative interviews (n = 16)	Medium-Low
Human Rights Watch Africa (1996) [40]	Legal	Rwanda	International Criminal Tribunal for Rwanda & state prosecution; training of police & judicial officers	Implementation description/data	Medium-Low
Mischkowski & Mlinarevic (2009) [62]	Legal	Yugoslavia	International Criminal Tribunal Yugoslavia &War Crimes Chamber-Bosnia & Herzegovina	Qualitative interviews (n = 49)	Medium
Nowrojee (2005) [41]	Legal	Rwanda	International Criminal Tribunal Rwanda	Qualitative interviews	Medium-Low
Denov (2006) [61]^a^	Legal	Sierra Leone	Truth and Reconciliation Commission & Special Court for Sierra Leone	Field visits & interviews	Medium-Low
Women’s Initiative for Gender Justice (2010) [63]	Legal	Global	International Criminal Court	Implementation description/data	Medium-Low

^a^Denov (2006) [61] is listed in both Livelihood and Legal interventions as it reports on case studies containing both elements

### Data synthesis

A series of matrices assisted to identify common elements and patterns. Given the paucity of detail in the studies (with weight of evidence at or below Medium-Low in 15 of the 20 studies reporting outcomes), only moderate analysis was possible on contextual factors. In recognition of this limitation we have labelled the project a ‘realist-informed’ review.

The review was registered with EPPI-Centre, a British academic body specialising in reviews of social and health interventions, with emphasis on low and middle-income countries (http://eppi.ioe.ac.uk/cms/).

## Results

**Fig. 1:**
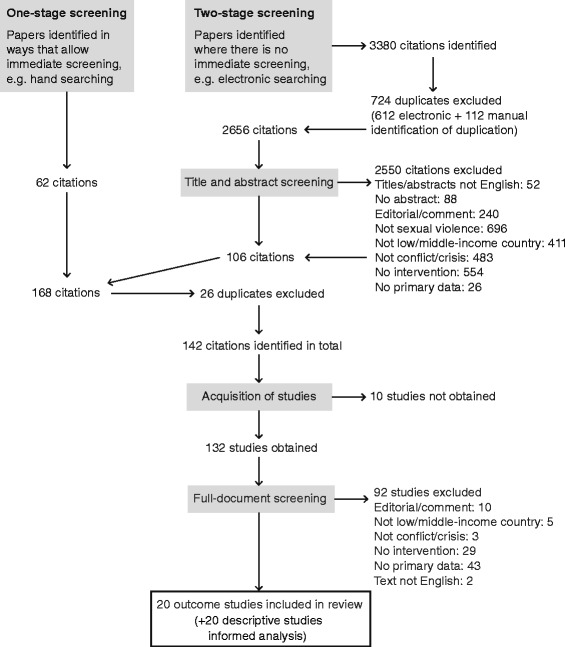
Filtering of search results

Results of the search strategy are reported in Fig. 1. Twenty studies were included: 11 identified from websites, one from a key informant and the remaining 8 through bibliographic databases. The studies were conducted in 17 countries predominantly in Africa with Rwanda being the country in which most studies were conducted (4). Nine studies addressed sexual violence which occurs in conflict settings, with 11 addressing post-conflict settings. No outcome studies addressed the disaster setting. 20 additional studies which reported on interventions, but which did not include outcomes informed the analysis. One of these was disaster focused [37]. Table 2 describes the included studies differentiated by intervention type. For the most part the studies reported on separate programs, although two included data on the Médecins Sans Frontières program in Congo [38, 39], and two others reported on the International Criminal Tribunal for Rwanda [40, 41].

The proposed mechanisms underpinning interventions developed by the team are first described, followed by the realist analysis. We initially proposed six mechanisms which, separately or in combination, might underpin interventions for sexual violence in conflict and crisis. These mechanisms relate mostly to the reasoning of the various actors involved [23], whether perpetrator, survivor or community, as is reflected in the names of each. Table 1 summarises the intervention types, proposed underpinning mechanisms and the associated outcomes.

### Proposed underpinning mechanisms

#### ‘Rape is risky’

Drawing on the criminological theory of deterrence [30], we propose ‘Rape is risky’ as the dominant mechanism for preventing sexual violence. According to this, sexual violence is less likely to occur as the risk of the perpetrator’s detection is increased through, for example, the presence of patrols or an increased likelihood that reports are taken seriously. The physically vulnerable and those least likely to be believed, such as children and those with a mental illness, are disproportionately targetted by sexual offenders [42–44] because the risk of their being detected by others is low. Although deterrence is often seen to be related only to legal responses, we propose that it is in fact relevant to all intervention types: for example increasing community willingness to take action makes sexual violence riskier for perpetrators. We theorised that a wide range of interventions can increase the perceived risk of rape, directly or indirectly.

#### ‘Rape is unacceptable’

This mechanism proposes that violence is reduced when potential offenders recognise that sexual violence or abuse is unacceptable and desist. This may result from training, community leadership or awareness activities. Examples may include disarmament, demobilisation and reintegration of participants returning home post-conflict with changed views, or well-monitored codes of conduct for combatants. There is emerging evidence that militarised sexual violence by insurgents and other combatants is not universal, particularly if strong leadership is exercised by commanders [45].

#### ‘There is help for this problem’

We suggest this mechanism is triggered when survivors of sexual violence become aware that services are available and that support or redress can be obtained. It may also operate through family or community members providing support to survivors, requesting interventions, or themselves sanctioning perpetrators. The first step towards this is awareness that services exist, which may also facilitate community recognition and naming of problems. The outcome of this mechanism is evidence that those who experience violence are aware of available survivor care services, which are an important conduit to formal reports to police and legal action. Ensuring survivors are aware of services is therefore critical [46].

#### ‘It’s safe to tell’

This mechanism is proposed to operate when survivors of sexual violence determine that they can safely report assaults or receive help without risk of punishment or sanction. The mechanism recognises that survivors will not report sexual violence to service providers if their confidentiality is not protected, or to authorities if they believe they are likely to be stigmatised or punished [46, 47]. Unless assaults can be safely reported, offenders will not be brought to account or deterred.

#### ‘We have rights’

Here individuals act in the belief that they are entitled to safety, protection or redress and are empowered to speak out against threatened or actual sexual violence. Unless individuals are aware of their right to refuse sex, to protest, to expect protection and to make decisions for themselves, and have these rights recognised, protective or accountability measures will not suffice to prevent sexual violence [48]. When this mechanism operates, we surmise that sexual violence is more likely to be reported and so poses risks to offenders.

#### ‘We can work together to address this problem’

This mechanism is proposed to operate where community leaders, members or agencies decide to work collaboratively with others to address sexual violence. Interventions involving empowerment, and where genuine community participation and changes in community norms are part of the context, show promise in reducing partner violence in low-income countries [49] and are suggested here to be relevant for all interventions. The outcome is that the community assists and protects survivors, at the same time as sanctioning offenders.

### Evidence of operation of mechanisms

In describing the results we focus on interventions where there was sufficient material to draw inferences or conclusions about the operation (or lack of operation) of mechanisms. We found nine studies that showed evidence of reduced risk or incidence of sexual violence. Multiple-component interventions tended to be associated with greater evidence of reduced risk or incidence and are presented first. This is followed by findings from single-component interventions, presented in the order of most promising outcome.

#### Multiple-component interventions

Four of the nine studies for which we found evidence of reduced risk or incidence [18] involved multiple-component interventions [38, 50–52]. Indicators of reduced risk reported in these studies included reduced incidence of cases and strong uptake of survivor care responses [38]; increased reporting and introduction of community-identified risk strategies [50]; high uptake of services (one of two sites only) [51]; and provision of stoves, removing the need to collect firewood [52].

The most common elements in the four multiple-component interventions with reduced risk or incidence were community mobilisation initiatives (all), systems and security initiatives (in three of four), and survivor care responses (in three of four). Crisis intervention teams established in a camp for Rwandan refugees in Tanzania are a good example of a multiple-component intervention [50]. This intervention was developed following community consultations conducted with separate age and gender groups. The teams trained community volunteers to provide a first-line support response to survivors and conducted awareness-raising activities, including discussion groups and a camp newsletter [50]. Firewood was also provided to households.

Systems and security components predominantly involved providing fuel alternatives and establishing firewood patrols. The mechanism proposed for such strategies was ‘Rape is risky’, which in these studies was identified as operating if the incidence of sexual violence was reduced when firewood patrols or fuel alternatives were present, consequently reducing opportunities for perpetrators to offend. This mechanism appeared to be operating in three of the multiple-component interventions, involving firewood distribution [50]; provision of ethanol stoves and fuel to 90 per cent of households [52]; and increased police presence [51]. The last of these studies reported that when men in the community were asked to collect firewood to protect women from risk of sexual violence, they resoundingly rejected it as inappropriate to their gender [51]. This example highlights the influence of cultural values and the importance of interventions which help shift social norms.

Although it is possible that the systems and security components on their own may have led to reduced risk of sexual violence, multiple-component interventions appear to be more effective than stand-alone interventions. Three other mechanisms were found to underpin different elements of these interventions. ‘There is help for this problem’ appeared to be operating, as indicated by the strong uptake of services by survivors. For example in the Kibondo refugee camp 1000 survivors of sexual violence accessed survivor care services in a 12-month period, compared to 23 in the neighbouring camp [50]. Strong uptake of services was found in four of five multiple-component interventions which provided survivor care [38, 50–53].

To use services, survivors must see them as safe. We postulated this to be a distinct mechanism, that is, ‘It’s safe to tell’. Initially we found it challenging to establish separate evidence for this mechanism, as distinct from ‘There is help for this problem’. However, their distinct nature was illustrated by the outcome of an Eritrean survivor care intervention that attempted to provide care to women assaulted during the country’s occupation [54]. The intervention was instigated in response to a request by community elders, but ultimately husbands and fathers prevented women using the service, fearing stigma and loss of marriage-price. The community was aware of the availability of the intervention so the mechanism ‘There is help for the problem’ was operating; however, ‘It’s safe to tell’ was not operating, evidenced by the study’s conclusion that survivors feared punishment if they used the service. This study reinforced the separate contribution of both mechanisms to successful implementation of services. Three multiple-component intervention studies with high service uptake had instituted specific measures to ensure confidentiality or anonymity. Two studies described a drop-in centre in a hospital [38, 51] and in a third services were provided ostensibly for ‘war trauma’ rather than specifying ‘sexual violence’, thus offering users a greater degree of protection [53]. These approaches reduced stigma and promoted service uptake, important for both encouraging reporting and reducing harm to survivors. Although survivor care responses do not directly reduce incidence of sexual violence, they allow the problem to be named and identified at community level. They may also reinforce the seriousness of the problem, encouraging reporting.

The fourth mechanism which we found to be operating in the multiple-component interventions was ‘We can work together to address this problem’*.* All four multiple-component interventions which showed indicators of reduced risk employed community mobilisation strategies. These included engagement of leaders; community bans on alcohol with curfew and night patrols [38]; community discussion and consultation [50, 52]; training community volunteers to provide the first response to survivors [50]; and awareness-raising activities [51].

One of the anticipated outcomes of this mechanism is that the community simultaneously assists and protects survivors while sanctioning offenders, signalling the unacceptability of sexual violence. Although limited data were reported on the specific impact of these strategies, community engagement appeared to be an important element in the impact of the multiple-component interventions.

These three mechanisms – ‘There is help for this problem’, ‘It’s safe to tell’ and ‘We can work together to address this problem’ – appeared necessary for the primary mechanism, ‘Rape is risky’, to bring about reduced risk and incidence, and proved to be central to desired outcomes in the stand-alone interventions as well.

#### Systems and security interventions

Systems and security interventions predominantly entailed firewood patrols or provision of fuel or alternative cooking equipment to households, as in the multiple-component interventions. Two of three studies using this strategy reported reduced incidence of sexual violence [55, 56]. A third study found that risk was lowered when women learnt to make fuel-efficient stoves, thus reducing the frequency of firewood collection [57].

While one study reported that this type of intervention led to reduced sexual violence during firewood collection, a corresponding *increase* in sexual violence in other camp locations was also reported [55]. The two interventions for which reduced incidence was reported, were accompanied by efforts to engage community members. In the Kenyan intervention, awareness-raising programs were held in schools and reporting systems were improved [56]. Similarly in Sudan, introducing committees to advise on the firewood patrols and provision of fuel was reported to build problem-solving skills among the community and trust towards the service provider delivering the intervention [57]. These findings suggest that the mechanism ‘We can work together to address this problem’ underpins successful outcomes for systems and security responses.

#### Personnel interventions

Personnel initiatives targeting sexual exploitation and abuse were also expected to operate through the mechanism ‘Rape is risky’*.* The two studies on personnel interventions described the introduction of zero-tolerance policies for sexual exploitation or abuse by peacekeepers in Haiti, Liberia [58], Kenya, Namibia and Thailand [59]. Only in the Liberian site was the policy effective where a comprehensive outreach campaign and action on a number of cases involving senior officers occurred [58]. By contrast, in Haiti a confidential hotline for reporting had not been promoted to the community and other methods for reporting were not confidential, putting those who made reports at risk of retaliation [58]. The second study similarly found lack of community consultation, little awareness of the policy and reporting mechanisms in the community, and low rates of reporting of incidents [59]. Both studies indicated a failure to inform communities and ensure the safety of survivors. These findings suggest that the same three mechanisms which were pivotal to multiple-component interventions, that is ‘There is help for this problem’, ‘It’s safe to tell’ and ‘We can work together to address this problem’, underpin personnel interventions as well.

#### Legal strategies

Our theoretical framework suggests that legal interventions may also operate through the ‘Rape is risky’ mechanism and this is in fact one of the classic rationales for prosecuting offenders of any crime. However, it would be difficult to find positive evidence of this link, given the length of criminal proceedings. Indeed, none of the legal interventions were found to deter sexual violence. In 5 of the 6 studies on legal strategies there was evidence that the mechanism was *not* operating in legal responses, with low rates of prosecution and conviction for sexual violence [40, 41, 60–62]. The exception was a study involving the International Criminal Court, which found that 6 of 10 matters before the Court had included charges for sexual violence. Yet, even in this study only 27 % of sexual violence applicants had been given leave to participate, indicating that the majority of sexual violence victims whose complaints make it as far as trial are still being turned away [63]. Other findings confirm that ‘It’s safe to tell’ was not operating in legal strategies. For example, women who testified in the Gacaca courts in Rwanda reported being re-traumatised by giving evidence and experiencing subsequent retaliation [60]. Under-reporting by sexual violence victims due to lack of protection and fear of stigma and retaliation was also reported in respect of the International Criminal Tribunal of Rwanda [40, 41]. Further survivors of sexual violence were unwilling to testify to the Sierra Leone Truth and Reconciliation Commission due to fear of reprisal [61]; and the trauma of testifying and a reported lack of confidentiality and dismissive treatment by investigators and prosecutors at the International Criminal Tribunal for Yugoslavia and War Crimes Chamber [62] also discouraged use of legal remedies. Taken together, these findings strongly suggest that for women who report assaults, it is not ‘safe to tell’, pointing to significant limitations in this arena.

#### Survivor care and livelihood interventions

Data indicating strong uptake of services suggest that the mechanisms ‘There is help for this problem’ and ‘It’s safe to tell’ were operating in two of the four survivor care initiatives. The Congolese post-rape medical and psychological support intervention treated 1115 women over 4 years [39] and the support groups in Afghanistan were attended by 137 women in a 17-month period [64]. Although neither of these studies contain data on the total number of *potential* service users, the numbers of women accessing the services indicate they were known of and seen as safe to use.

There was little other material in the identified studies reporting survivor care and livelihood strategies that allowed analysis of other mechanisms.

### Mechanisms not supported by the data

Two of our proposed mechanisms were not supported by data from the included studies. The first of these, ‘Rape is unacceptable’, proposed that offenders or potential offenders desist as a result of attitude change. No studies of the impact of men’s behaviour change programs were identified in our search. However since this date a pilot trial of a 16 week men’s discussion group aimed at preventing intimate partner violence in conflict zones in Cote d’Ivoire, found changes in men’s reported ability to control hostility and manage conflict but not significant changes to reported physical or sexual violence [65]. As a result capacity to determine whether this mechanism underpins effective interventions is limited. The second mechanism for which we found no evidence was ‘We have rights’. We acknowledge that in the crisis context it would be challenging for studies to directly explore decisions about whether to take action with sexual assault survivors, which would be necessary to test the presence or absence of this mechanism.

### An unanticipated mechanism

A mechanism which we had not anticipated was ‘We already have ways to address this problem’, which we defined as recognition by those providing interventions of pre-existing capacity or systems within the community to address sexual violence. It was evident when community members used strategies to address sexual violence which derived from existing cultural practices. Examples included mutual support groups in Rwanda which drew on traditional concepts of *kwihanga* (withstanding), *kwongera kubaho* (living again), and *gukomeza ubuzima* (continuing life/health) to enable women to make meaning from their experiences and build resilience [66].

Two other studies provided further support for this mechanism. A program for adolescents recruited into the Lord’s Resistance Army who were both survivors and perpetrators of sexual violence employed traditional healing rituals. Performed by elders and involving traditional music, dance and cleansing rituals, this program aimed to exonerate and remove shame [67]. In a Kenyan refugee camp, survivors used a hierarchy of responses to deal with gender-based violence, first attempting to resolve it themselves or with family, then through elders, with external agencies as a last resort [68]. The author reported significant community resistance to the ‘gender equality lens’ of service provision by international NGOs, seen by men in the community as “harming the family and community” [68], and noted the potential of community-derived programs. The mechanism ‘We already have ways to address this problem’ recognises that existing strategies may be more likely to be embraced by communities, in contrast to interventions reflecting western ideas, which may be seen as divisive [46, 54].

## Discussion

Adopting a realist approach has enabled us to consider whether interventions work or not, and what mechanisms may have contributed to this. Although data was limited, our preliminary hypothesising and analysis strongly supported three proposed mechanisms as underpinning effective interventions for conflict and crisis-related sexual violence: ‘We can work together to address this problem’, ‘There is help for this problem’, and ‘It’s safe to tell’. Surprisingly, these three mechanisms appeared to be central across diverse types of intervention, including multiple-component interventions. For example, there is evidence of the mechanism ‘We can work together’ in systems and security strategies. Similarly, personnel interventions appear to require knowledge of service availability and safe reporting. Further, it appears that having these mechanisms operating simultaneously contributes to greater likelihood of success. Although we assumed that perpetrators’ recognition that ‘Rape is risky’ was a central mechanism in deterring sexual violence, the evidence suggests that this mechanism operated only in interventions focused on gathering firewood and providing alternative fuels. The other interventions identified in this review did little to deter sexual violence. The results also suggest that in order for this mechanism to operate, the other three mechanisms may also be required to support its operation.

### Alignment of findings with other research

Support for the centrality of the three mechanisms ‘We can work together to address this problem’, ‘There is help for this problem’, and ‘It’s safe to tell’ are confirmed by other recent studies. A community prevention program implemented in post-conflict settings in South Sudan, Uganda, Thailand, Liberia and Rwanda, used local production and playback of videos produced to address local concerns in relation to gender-based violence, including engagement of local community leaders to provide strong support for the project [69]. The qualitative study suggested that community changes included; cultural norms permitting early girl marriages; recognition of forced marriage and gendered violence as a punishable crime; and an increased uptake of GBV services; these successes mainly as a result of the inclusion and strong support of the local community leaders [69]. Similarly the introduction of a mobile clinic in South Kivu, DRC was supported by first building local partnerships with existing health administrators, and consultation with local experts at each of the six sites, promoting the service through community health workers, resulting in strong uptake of services; and finally, locating the service in existing health services and extending it to those who had not experienced sexual violence to protect anonymity [70].

The failure of the ‘Rape is risky’ mechanism in most interventions is supported by evidence that weak legal and social sanctions are risks for sexual violence [71]. Without apprehension and punishment of offenders, little can be expected in terms of prevention. The fact that this mechanism was triggered in only a few interventions is consistent with other literature. For example, prosecutions of police and peacekeepers for sexual violence was a deterrent only in the rare instances in which high-ranking commanders were held to account [72]. It is essential that where legal action is taken, that determinations do not further victimize survivors with separate evidence that forced marriages to the perpetrators or fines been paid to the family for harm done can serve to expose survivors to further abuse and stigma [73].

The mechanism ‘It’s safe to tell’ fits with Ho and Pavlish’s [48] emphasis on the critical role of empowering women in refugee camps and removing barriers to recognising and demanding their rights in relation to gender-based violence. Further evidence of the importance of this mechanism is found in recent research in conflict and post-conflict settings which found that low and late attendance at health services for care after sexual assault was attributable in part due to fear experienced by survivors [74]. The same study also provided support for ‘There is help for this problem,’ with evidence that many referrals to the service occurred through the work of “counsellor mothers” a network of trained community advocates who provided localised awareness of the availability of the service including making direct referrals. The South Kivu outreach program identified that women under 20 years were not accessing the clinics, so for this group it was not ‘safe to tell’. Consultation revealed this was due to young women’s fears of damage to marriage prospects if they were identified attending clinics and by default as being sexually active. Additional strategies were being explored to address this mechanism through for example integrating health care with socio-economic interventions [70].

The need for the mechanism ‘We can work together to address this problem’ has also been noted elsewhere, with findings that building and nurturing trust with civil society organisations [75] and consultation with all segments of the community are key responses to gendered violence [76]. Without consultation, programs for conflict-related sexual violence risk being ill-adapted to local traditions and culture, and are likely to be rejected by the women they are designed to assist [6]. The central idea of the mechanism ‘We already have ways to address this problem’ has also previously been expressed, for example through recognition that women already engage in community-based initiatives relevant to Security Council Resolution 1325 [77] and is supported by findings of the importance of indigenous expertise in developing sustainable strategies in conflict and post-conflict settings [70].

### Strengths and limitations of the review

This is the first review adopting a realist frame in this important but under-studied area. The approach enabled us to investigate the mechanisms underpinning the intervention types and their contribution to the success of the interventions. This provides a strong base for more focused research in this area. The review was, however, limited by the low weight of evidence in most of the identified studies, due to factors such as small sample sizes, self-selection of participants, reliance on qualitative data, lack of robust outcome measures and limited data enabling identification of relevant contextual factors. We may have overlooked some relevant studies because their key words, title or abstract do not explicitly refer to addressing sexual violence or to conflict, post-conflict, crisis or refugee settings, or were in languages other than English.

### Implications for practice, policy and research

This work reinforces the importance of integrated, multiple-component interventions to address sexual violence in conflict and crisis, which may be supported by the operation of three key mechanisms, ‘There is help for this problem’, ‘It’s safe to tell’ and ‘We can work together to address this problem’. The analysis also highlights the value of the same mechanisms in diverse stand-alone interventions, that is, in systems and security responses, personnel interventions, legal responses, and survivor care services. Despite the distinct differences between these types of intervention, the same mechanisms appear to be important to their successful operation. Legal strategies appeared to be lacking in their capacity to provide safety to survivor witnesses, and this absence of the mechanism (‘It’s safe to tell’) would seem to support its place in a successful intervention. For service providers and policy makers designing new programs, the results point to the merit of incorporating these mechanisms to maximise safe uptake of services and ultimately deterrence of sexual violence. The mechanism ‘We already have ways to address this problem’ points to the need to determine whether communities already have strategies in place to address sexual violence which may be preferred or that can be incorporated into introduced interventions, prior to their commencement. This would be respectful of community values and capacity and would potentially improve the acceptability of interventions.

Further research is required to fully test the proposed mechanisms. This requires robust evaluation of multiple-component programming among other types of intervention, with attention to gathering and reporting critical contextual information. Careful development and rigorous evaluation of such studies will enable the mechanisms proposed here to be validated, refuted, extended or refined.

## Conclusion

Realist approaches offer opportunities to identify underlying mechanisms that are central to the success or failure of programs, recognising the challenges which will prevail in undertaking research in conflict settings and drawing more deeply on existing research. Such findings can then be built into the design of interventions and delivery of programs to reduce conflict and crisis-related sexual violence.

## Abbreviations

CMO: Context, mechanism and outcome
GBV: Gender-based violence
NGO: Non-government organisation

## Footnotes

1 Medline (PubMed on Ovid), CINAHL, PsycInfo, PAIS, Global Health, ASSIA, Gender studies, Violence & Abuse Abstracts, Wageneingen University Disaster Studies, ProQuest Dissertations & Abstracts, Lexis-Nexis, UNICEF Children in armed conflict, GDNet Knowledge Base, African Journals Online, 3ie database of impact evaluations, Bibliomap & TRoPHI (EPPI-Centre), World Health Organization Library (WHOLIS), EBM Reviews, Cochrane Database of Systematic Reviews, Johanna Briggs systematic review and Campbell Collaboration databases.

2 As an example the search terms from Medline were as follows: “abused women” [text words] OR “abused woman”[tw] OR “forced sex”[tw] OR “enforced sex”[tw] OR GBV[tw] OR “gender based violence”[tw] OR rape[tw] OR raped[tw] OR rapist[tw] OR raping[tw] OR “sexual abuse”[tw] OR “sexual coercion”[tw] OR “sexual violence”[tw] OR “sexual assault”[tw] OR “sexual exploitation”[tw] OR “sexual slavery”[tw] OR “violence against women”[tw] OR “unwanted sex”[tw] OR “unlawful sex”[tw] OR “sexual exploitation and abuse”[tw] “militarised sexual violence”[tw] OR “forced pregnancy”[tw] OR “enforced pregnancy” [tw] AND “armed conflict”[tw] OR “armed incursion”[tw] OR “post conflict”[tw] OR “human security”[tw] OR “war zone”[tw] OR coup[tw] OR invasion[tw] OR insurrection[tw] OR “peace keeping”[tw] OR “peace building”[tw] OR “child soldiers”[tw] OR “boy soldiers”[tw] OR “internally displaced persons”[tw] OR “displaced populations”[tw] OR “displaced persons”[tw] OR “refugee camps”[tw] OR “humanitarian response”[tw] OR “humanitarian assistance”[tw] OR “humanitarian crisis”[tw] OR “humanitarian crises”[tw] OR “post-crisis” [tw] OR “post-crises”[tw] OR war[mesh heading] OR Refugees [mh] OR Disasters [Mesh:noexp] OR Disaster Planning [mh] OR Mass Casualty Incidents[mh] OR Relief Work[mh] OR Rescue Work[mh] OR Avalanches[mh] OR Earthquakes[mh] OR Landslides[mh] OR Tidal Waves[mh] OR Tsunamis[mh] OR Volcanic Eruptions[mh] OR Fires[mh] OR Cyclonic Storms[mh] OR Floods[mh].
